# The case for neuregulin-1 as a clinical treatment for stroke

**DOI:** 10.3389/fncel.2024.1325630

**Published:** 2024-04-04

**Authors:** Jessica M. Noll, Arya A. Sherafat, Gregory D. Ford, Byron D. Ford

**Affiliations:** ^1^Division of Biomedical Sciences, University of California-Riverside School of Medicine, Riverside, CA, United States; ^2^Nanostring Technologies, Seattle, WA, United States; ^3^Southern University-New Orleans, New Orleans, LA, United States; ^4^Department of Anatomy, Howard University College of Medicine, Washington, DC, United States

**Keywords:** clinical trial, excitotoxicity, inflammation, ischemia, neuregulin, neuroprotection, oxidative stress, stroke

## Abstract

Ischemic stroke is the leading cause of serious long-term disability and the 5th leading cause of death in the United States. Revascularization of the occluded cerebral artery, either by thrombolysis or endovascular thrombectomy, is the only effective, clinically-approved stroke therapy. Several potentially neuroprotective agents, including glutamate antagonists, anti-inflammatory compounds and free radical scavenging agents were shown to be effective neuroprotectants in preclinical animal models of brain ischemia. However, these compounds did not demonstrate efficacy in clinical trials with human patients following stroke. Proposed reasons for the translational failure include an insufficient understanding on the cellular and molecular pathophysiology of ischemic stroke, lack of alignment between preclinical and clinical studies and inappropriate design of clinical trials based on the preclinical findings. Therefore, novel neuroprotective treatments must be developed based on a clearer understanding of the complex spatiotemporal mechanisms of ischemic stroke and with proper clinical trial design based on the preclinical findings from specific animal models of stroke. We and others have demonstrated the clinical potential for neuregulin-1 (NRG-1) in preclinical stroke studies. NRG-1 significantly reduced ischemia-induced neuronal death, neuroinflammation and oxidative stress in rodent stroke models with a therapeutic window of >13 h. Clinically, NRG-1 was shown to be safe in human patients and improved cardiac function in multisite phase II studies for heart failure. This review summarizes previous stroke clinical candidates and provides evidence that NRG-1 represents a novel, safe, neuroprotective strategy that has potential therapeutic value in treating individuals after acute ischemic stroke.

## 1 Introduction

Stroke remains one of the most devastating diseases in modern healthcare. Previously ranked as the third leading cause of death, stroke has since decreased to fifth but maintains first in cause of adult disability in the United States (U.S.) (Adams et al., 2007; Powers et al., 2015; Benjamin et al., 2017; Tsao et al., 2023; Martin et al., 2024). There are two major classifications of stroke: ischemic, which is the occlusion or blockage of an artery within the brain, and hemorrhagic, which is rupture of an artery within the brain and subsequent bleeding into the surrounding tissue. According to recent reports from the American Heart Association (AHA), the global incidence of stroke was 11.71 million in 2020 with 7.59 million as ischemic strokes. There were 3.48 million deaths from ischemic stroke and 0.35 million deaths from hemorrhagic stroke worldwide (Tsao et al., 2023; Martin et al., 2024). From 2010 to 2020, the age-adjusted stroke mortality rate in the US (38.8 per 100,000) decreased 0.8% and the actual number of stroke deaths increased 23.8%.

Ischemic stroke is caused by occlusion of blood flow to the brain resulting in localized areas of neuronal death called infarcts. This initial area of brain injury (the infarct core) occurs within minutes and is characterized by low cerebral blood flow, energy failure, excitotoxicity, and edema [for reviews, Barone and Feuerstein, 1999; Dirnagl et al., 1999; Wu et al., 2018]. Ischemia results in cellular energy depletion, neuronal overstimulation and excessive glutamate release from neurons leading to glutamate-induced excitotoxicity and necrotic neuronal death. Necrotic neurons in the ischemic core produce danger-associated molecular patterns (DAMPs) which are deemed as alarm signals by the innate immune system and lead to a delayed apoptotic neuronal death (Patel, 2018).

Neuronal death in the infarct core is believed to be irreversible and triggers the production of inflammatory molecules and oxidative stress in neurons, glia, and in the cerebral vasculature, which endangers brain cells in a larger, surrounding area known as the ischemic penumbra (Stoll et al., 1998; Dirnagl et al., 1999; del Zoppo et al., 2000; Iadecola and Alexander, 2001; Touzani et al., 2001; Iadecola and Anrather, 2011). In the penumbra blood flow is reduced but neurons can survive for several hours following stroke onset, suggesting that the therapeutic window for neuroprotective stroke treatment may be quite prolonged. In the penumbra, inflammatory cytokines, such as interleukin-1β (IL-1β), are induced in the ischemic brain of animal models and in human stroke as well. These cytokines increase the expression of downstream pro-inflammatory molecules, oxidative stress genes and adhesion molecules that promote delayed neuronal injury in the penumbra. Interventions that inactivate cytokines or that block cytokine receptors reduce ischemic damage in animal models of stroke.

To date, only one drug is FDA-approved for acute ischemic stroke treatment: tissue plasminogen activator (tPA), also known as alteplase (The National Institute of Neurological Disorders and Stroke rt-PA Stroke Study Group, 1995; Kleindorfer et al., 2008; Lees et al., 2010; Powers et al., 2015). The FDA approved the use of tPA for the treatment of acute ischemic stroke in 1995, but not without considerable restrictions of use due to side effects. Early clinical trials exhibited high rates of intracerebral hemorrhage but found clinical benefit when administered within 3 h of stroke (The National Institute of Neurological Disorders and Stroke rt-PA Stroke Study Group, 1995). A later study utilized data from four major stroke clinical studies with tPA: ECASS III, Alteplase Thrombolysis for Acute Noninterventional Therapy in Ischemic Stroke (ATLANTIS) Neurological Disorders and Stroke (NINDS), Echoplanar Imaging Thrombolytic Evaluation Trial (EPITHET) and determined that tPA yielded beneficial outcomes when given up to 4.5 h but not beyond, and the earlier patients are treated, the better the outcome (Lees et al., 2010). Due to the hemorrhagic risk, only 1.8–2.1% of U.S. ischemic stroke patients are treated with tPA per year with only an approximate annual 0.04–0.09% increase in this treatment number (Kleindorfer et al., 2008). While thrombolytic agents that break down existing clots can result in neuroprotection, these agents primarily target the cerebral vasculature, rather than neuronal cells, and so are considered indirect neuroprotectants.

Several potentially neuroprotective agents, including glutamate antagonists, anti-inflammatory compounds and free radical scavenging agents were shown to be effective neuroprotectants in preclinical animal models of ischemia (Muir and Grosset, 1999; O'Collins et al., 2006; Fisher et al., 2009; Saver et al., 2009; Chen and Wang, 2016; Bosetti et al., 2017). Unfortunately, after nearly 200 clinical trials, all attempts at neuroprotection for ischemic stroke clinically have failed. The Stroke Treatment Academic Industry Roundtable (STAIR) first met and published recommendations in 1999 specifically to address stroke preclinical studies intended to increase success of stroke therapeutics brought to clinical trials (Fisher et al., 2009; Albers et al., 2011; Lapchak et al., 2013). STAIR meetings consist of academic physicians, industry representatives, and regulators that have met multiple times since the initial 1999 meeting to provide updates to the STAIR criteria, adapting to continuing stroke clinical trial outcomes (Fisher et al., 2009; Saver et al., 2009; Albers et al., 2011). The STAIR criteria were created as preclinical study recommendations with the purpose of enhancing the success of stroke treatments, neuroprotective specifically. Current STAIR recommendations and updates designed to be utilized as an outline for preclinical stroke studies are summarized below (Fisher et al., 2009; Lapchak et al., 2013):

1. Adequate dose–response curve: The minimum and maximum tolerated dose should be defined as well as proof that the target organ is reached. There should also be a “reasonable prospect” of clinical benefit within this dose in human administration.
2. Define the time window in a well-characterized model: Address the therapeutic window relevant to your model in relation to humans, specifically regarding thrombolytic and neuroprotective drugs.
3. Randomized and blinded studies:

- Report animals excluded from analysis
- Allocation concealment occur and should be described
- Conduct full power analysis and sample size calculations

4. Histological and functional outcomes assessed acutely and long-term (update): both histological and behavioral outcomes should be assessed, ensuring studies take place at least 2–3 weeks after stroke onset to demonstrate sustained benefit.
5. Permanent occlusion then transient in most cases (with physiological monitoring update): During both permanent and transient occlusion, ensure basic physiological parameters are being monitored (blood pressure, temperature, blood gases, blood glucose, etc.) with important attention to cerebral blood flow via Doppler.
6. Initial rodent studies, then consider gyrencephalic species (Multiple species update): Establish treatment efficacy in at least 2 species. Rodents and rabbits can be initial tests, but secondary species is ideally gyrencephalic (primate, cat, etc.).
7. Efficacy in two or more laboratories (Reproducibility update):

- Eliminate randomization and assessment bias
- Define inclusion/exclusion criteria
- Further studies in females, aged animals, and co-morbid conditions such as hypertension, diabetes, and hypercholesterolemia

8. Disclose potential conflicts of interest
9. Consideration of route of administration (to administer intravenous or not)
10. Consideration of sex differences

STAIR has released additional recommendations on multiple focus areas including: rapid administration, increasing the therapeutic window, and adjuvant treatment (Fisher et al., 2009; Saver et al., 2009; Albers et al., 2011; Lapchak et al., 2013; Jovin et al., 2016). When designing preclinical trials for a potential stroke therapeutic, it is suggested that one should address each of the 10 STAIR recommendations listed above as well as additional STAIR recommendations directly related to the specific study focus.

This review will: 1) discuss previous stroke neuroprotective drugs tested unsuccessfully clinical trials; 2) introduce NRG-1 as a novel strategy to treat ischemic stroke; and 3) describe lessons learned from the design of successful stroke clinical trials (e.g., tPA and endovascular thrombectomy (EVT) to inform clinical trial design for NRG-1. These findings could support the development of clinical studies using NRG-1 alone or in conjunction with other therapies, such as thrombolysis and EVT, for the treatment of patients with acute stroke.

## 2 Previous preclinical and clinical stroke neuroprotection studies

### 2.1 Excitotoxicity/glutamate antagonists

The well-established role of excess glutamate in neuronal death, excitotoxicity, after ischemia and traumatic brain injury (TBI) led to the theory of neuroprotection via blocking the effects of glutamate excitotoxicity (Simon et al., 1984; Novelli et al., 1988; Faden et al., 1989). Excitotoxicity neuroprotective agents primarily target the *N*-methyl-D-aspartate (NMDA) glutamate receptor due to its demonstrated role in glutamate-induced cell death (Novelli et al., 1988). However, NMDA receptor antagonists have left a wake of failed clinical trials including, Selfotel, Aptiganel, Eliprodil, Licostinel, and Gavestinel. Many were tested in TBI and stroke clinical trials from Phase I to Phase III. Animal trials exhibited positive neuroprotective results, but most of the studies administered the drug prior to injury and did not consider a clinically relevant therapeutic window for stroke (Meldrum, 1990; Birmingham, 2002; Ikonomidou and Turski, 2002). In addition, later animal studies indicated that the NMDA receptor antagonist, MK-801, did not provide direct neuroprotection, but rather created a hypothermic environment that resulted in neuronal sparing (Buchan and Pulsinelli, 1990; Buchan and Pulsinelli, 1991). As a result, failure to test a clinically relevant therapeutic window and understand the chosen drug’s mechanism of action led to poorly designed clinical trials for glutamate antagonists. Trials from Phase I-III with all NMDA antagonist resulted in: 1) no benefit seen; 2) speculated or confirmed increase in severe adverse effects and 3) speculated or confirmed increase in mortality (Albers et al., 1999; Morris et al., 1999; Davis et al., 2000; Albers et al., 2001; Sacco et al., 2001; Birmingham, 2002).

### 2.2 Anti-inflammatory agents

A significant and prolonged inflammatory response is initiated during ischemic injury, beginning from core damage, and believed to play an important role in the progression of penumbral damage. Microglia are activated early on in the inflammatory response, leading to the release of reactive oxygen species, nitric oxide, cytokines [such as IL-1β and tumor necrosis factor-α, (TNF-α)] and chemokines, which later contribute to continued inflammation via invading immune cells such as leukocytes (Liu et al., 1993; Buttini et al., 1994; Betz et al., 1996; Barone et al., 1997; Jin et al., 2010; Graeber et al., 2011). Neuroprotection became a target for anti-inflammatory agents by focusing on the evolution of the inflammatory response in ischemia, including, neutrophil inhibition, anti-ICAM-1, IL-1 receptor inhibition, CD11b/CD18 inhibition (targeting neutrophil adhesion), and induction of E-selectin tolerance among others. Agents that were involved in stroke clinical studies include Enlimomab (anti-ICAM-1), neutrophil inhibitory factor, Interleukin-1 receptor antagonist (IL-1ra), and minocycline. In preclinical rodent studies, ICAM-1 knockout animals demonstrated reduced infarct volume in transient ischemic stroke model, but not in a permanent model, showing that neuroprotection is only present when followed by reperfusion (Connolly et al., 1996; Kitagawa et al., 1998; Prestigiacomo et al., 1999; Soriano et al., 1999; Kanemoto et al., 2002). Rats treated with a combination of tPA and anti-ICAM-1 or anti-CD18 antibody demonstrated reduced infarct and neurological deficit compared to tPA treatment alone when treated <4 h after reperfusion (Zhang et al., 1999a,b). However, Enlimomab studies for stroke were terminated at Phase III trials due to worse long-term outcome seen at 90 days, a higher mortality, and more severe adverse effects with treatment (Investigators, 2001).

UK-279,276 (neutrophil inhibitory factor) successfully demonstrated reduced neutrophil infiltration and infarct volume in a transient rat middle cerebral artery occlusion (MCAO) stroke model and in combination with tPA in a thromboembolic rat stroke model when administered <4 h (Jiang et al., 1998; Zhang et al., 2003). Acute Stroke Therapy by Inhibition of Neutrophils (ASTIN) clinical studies with neutrophil inhibitory factor for stroke were terminated at Phase II as patient recovery did not improve but no serious side effects were seen (Krams et al., 2003; Grieve and Krams, 2005; del Zoppo, 2010). IL-1ra preclinical trials repeatedly demonstrate neuroprotection in rat stroke models in multiple administrative methods (i.v. bolus, i.v. infusion, and s.c.) with sustained infarct reduction at 24 h and 7 days post-injury (dpi) with respect to treatment administration <3 h (Loddick and Rothwell, 1996; Relton et al., 1996; Mulcahy et al., 2003; Clark et al., 2008; Greenhalgh et al., 2010). IL-1ra clinical trials were first associated with no clinical benefit and were terminated after Phase II (del Zoppo, 2010; Smith et al., 2018). It was subsequently shown that initial rodent models lacked studies using systemic administration, delayed treatment, and comorbid animal studies (Sobowale et al., 2016).

Minocycline is a tetracycline antibiotic that has demonstrated anti-inflammatory, anti-apoptotic, and neuroprotective effects in animal stroke models leading to multiple clinical trials. In acute treatment, minocycline treatment demonstrated significant reduction of infarct size and inflammation in the rat ischemia model when administered <5 h (Wang et al., 2003; Xu et al., 2004; Nagel et al., 2008; Fagan et al., 2010). Delayed and prolonged treatment, beginning at 24 h and continuing for 14 days, improved neurological functioning and survival while preventing ischemic brain tissue atrophy (Hayakawa et al., 2008). This effect was supported when treatment was delayed to 4 dpi and prolonged to 4 weeks, with demonstration of significant decrease in activated microglia (Hewlett and Corbett, 2006; Liu et al., 2007). Additionally, minocycline demonstrated success when used in combination treatment with tPA, including reducing tPA related hemorrhage, MMP-9 levels, and blood–brain barrier (BBB) injury (Murata et al., 2008; Machado et al., 2009). Minocycline was successfully brought to clinical trials for ischemic stroke as oral monotherapy in 2007 (Lampl et al., 2007). This trial had a therapeutic time window of 6–24 h and measured endpoints as neurological recovery at 7, 30, and 90 days on the modified Rankin Scale (mRS), Barthel Index, and NIH stroke scale (NIHSS) (Lampl et al., 2007). Results found benefit in using the NIH Stroke Scale (NIHSS) scale at 1-day, Modified Rankin Scale (mRS) at 2 days, and Barthel Index at 7 days. No difference was determined related to ischemic location, magnitude of focal deficits, or medical cause. However, there was no difference in mortality, recurrent strokes, or hemorrhagic transformations. A later dose study in 2010 aimed to determine both the dose-limiting toxicity of minocycline and its safety when administered with tPA (Fagan et al., 2010). This study found that minocycline was well-tolerated intravenously when administered with tPA with mild adverse effects, up to 10 mg/kg, and no cases of severe intracerebral hemorrhage were observed. A subsequent study also showed that patients with acute ischemic stroke had significantly better outcome with minocycline treatment as compared with those administered placebo (Padma Srivastava et al., 2012). However, a later pilot study of a small sample of acute stroke patients showed that intravenous minocycline was safe but not efficacious (Kohler et al., 2013) and during a recent multisite clinical trial for minocycline, the data and safety monitoring board recommended ending the trial for futility after determining that it was highly unlikely for minocycline to show significant efficacy over placebo in improving functional outcomes at 90 days (Singh et al., 2019).

### 2.3 Oxidative stress/free radicals

Following ischemic brain injury, large amounts of reactive oxygen free radicals are produced and released, leading to progressive cellular damage (Cheng and Sun, 1994). Drugs have been developed in hope of counteracting this process as free-radical scavengers. Three drugs will be discussed under this category: Tirilazad, Citicoline, and NXY-059. A meta-analysis reviewed the study quality and study outcome measurements of 18 pre-clinical studies involving neuroprotection of Tirilazad in animal models of stroke (Sena et al., 2007). This analysis found outcome measurements were infarct volume and/or neurological score with average findings that Tirilazad reduced volume by 29.2% and improved neurobehavioral score by 48.1%. The studies exhibited that Tirilazad did demonstrate efficacy in animal models, but with study quality median of 5/10, this conclusion presents possible presence of bias (Sena et al., 2007). Tirilazad was brought to stroke clinical trials in the U.S. because studies in Europe, Australia, and New Zealand reported reduced mortality and good recovery with treatment (Haley et al., 1997). However, when Tirilazad underwent clinical trials in the U.S., it resulted in a considerable lack of efficacy (Haley et al., 1997; Haley, 1998). This was suspected to be due to a low dose in female patients. A follow-up study with higher doses including male and female patients was done, but was prematurely terminated due to safety questions in a parallel study (Haley, 1998). Results were still published from this study and determined that the drug was well-tolerated with no evidence of harm, but consideration that there may be a difference in patient admission characteristics, management protocols, or use of anticonvulsant medication that could have led to variable results (Haley et al., 1997; Haley, 1998).

Citicoline followed a similar pattern. A meta-analysis assessed 14 studies utilizing citicoline as a neuroprotectant in animal stroke models (Bustamante et al., 2012). This analysis found on average that citicoline reduced infarct volume by 27.8% and improved neurological deficit by 20.2% in ischemic occlusive stroke models with higher efficacy in proximal occlusion and in combination treatment with tPA (Bustamante et al., 2012). However, this analysis noted that these studies had an absence of co-morbidities, females, old animals, or strain differences, which notes failure to fulfill STAIR criteria (Bustamante et al., 2012). This drug demonstrated beneficial effects in both rodent and non-U.S. clinical stroke trials with significant functional and neurological improvement (Clark et al., 1997). However, a follow-up trial in the U.S. determined that citicoline was safe but ineffective for improving the outcome for mild stroke patients and suggested it be tested in moderate to severe stroke patients (Clark et al., 1999). A large, international trial was conducted to pool the results found from various trials with treatment of patients with moderate to severe ischemic stroke considering citicoline was approved in multiple countries but has yet to show significant efficacy (Dávalos et al., 2012). This trial was terminated at the third interim as citicoline was still not found to be efficacious in treatment of moderate to severe ischemic stroke (Dávalos et al., 2012).

Disodium 2,4-disulfophenyl-N-tert-butylnitrone (NXY-059) acts as a free radical trap that demonstrated neuroprotection following cerebral ischemia and reduced infarct volume in animal models but failed to follow-through in clinical trials. Initially, NXY-059 was hailed as a stroke neuroprotectant success, fulfilling significant results in pre-clinical STAIR criteria including BBB permeability, efficacy in two or more laboratories, neuroprotection, functional improvement, functionally adjuvant with tPA, and randomized, blinded studies. The first clinical trial with NXY-059 (SAINT I) significantly improved neurological function 3 months following stroke. However, the subsequent SAINT II trial showed no functional benefit of NXY-059. No significant sustained improvement in cognitive function, reduction of infarct size, or reduction of hemorrhage from tPA adjuvant treatment was exhibited (Shuaib et al., 2007; Bath et al., 2009). Closer analysis demonstrates that NXY-059 did not fulfill the STAIR criteria to the extent many believed and should have been evaluated more critically before advancing into clinical trials. Initial rodent studies utilizing the MCAO stroke model were found not to record the cerebral blood flow (CBF) during stroke via doppler. These studies displayed a mean average of 25% neuroprotection and approximately 50% of animals did not have a stroke, but results were still included as “neuroprotection” (Ginsberg, 2007; Savitz, 2007). Neurological improvements, although seen, were not sustained at 48 h after reperfusion, which does not fulfill the STAIR criteria (Savitz, 2007). In studies using the marmoset, NXY-059 treatment was administered 5 min after occlusion and preclinical animal models were performed primarily within one academic laboratory (Savitz, 2007). The findings showed a 28% reduction in overall infarct size that was not statistically significant, though neurological improvement was noted.

### 2.4 Nerinetide (NA-1)

Nerinetide (also known as TAT-NR2B9c and NA-1) is a neuroprotective peptide that ameliorates neuronal excitotoxic damage by preventing the activation of neuronal nitric oxide synthase (nNOS) which reduces overstimulation of NMDA receptors (Ballarin and Tymianski, 2018). In rat and mouse permanent and transient MCAO stroke models, Nerinetide reduced infarct volume and restored motor functions (Sun et al., 2008; Zhou et al., 2010; Bach et al., 2012). Nerinetide was also shown to reduce infarct volumes and preserved neurological function in a nonhuman primate cynomolgus macaque MCAO model (Cook et al., 2012a) and reduced the number and the volume of micro strokes in macaques injected with the polystyrene spheres (Cook et al., 2012b). In a Phase II clinical trial (ENACT; NCT00728182) patients were given 2.6 mg/kg of nerinetide immediately after the aneurysm repair and found that the number of lesions was significantly reduced by nerinetide treatment, but with no changes to lesion volume (Hill et al., 2012). In a Phase III clinical trial for nerinetide (ESCAPE-NA1; NCT02930018) efficacy in ~1,100 patients experiencing ischemic stroke undergoing rapid EVT was examined (Hill et al., 2020). Nerinetide was administered within 60 min from imaging and randomization. Nerinetide did not improve the clinical outcomes after EVT compared with patients receiving placebo. However, there was a promising signal of potential efficacy in the subgroup of patients who were not treated with t-PA.

## 3 Rationale for neuregulin-1 clinical studies for stroke

Neuregulins are a family of structurally related proteins that have diverse functions in the nervous system and have shown promise in treating stroke. Neuregulin-1 (NRG-1) belongs to a family of multipotent neuroprotective and anti-inflammatory growth factors that include acetylcholine receptor inducing activities (ARIAs), glial growth factors (GGFs), heregulins and neu differentiation factors (NDFs) (Holmes et al., 1992; Wen et al., 1992; Falls et al., 1993; Marchionni et al., 1993; Ho et al., 1995). The effects of NRG-1 are mediated by erbB tyrosine kinase receptors which include erbB2, erbB3 and erbB4 (Meyer et al., 1997). NRG-1 acts through homo-or heterodimerization of erbB receptors. The erbB receptors become phosphorylated upon ligand binding and activate a variety of signal transduction pathways including mitogen-activated protein kinase (MAPK), phosphatidylinositol 3-kinase (PI3K) and cyclin-dependent kinase-5 (CDK5) (Li et al., 2003; Croslan et al., 2008; Sawe et al., 2008; Cui et al., 2013, 2023). Studies from our laboratory showed that neuroprotection by NRG-1 can be mediated by the PI3K/Akt signaling pathway. NRG-1 prevented neuronal death in an *in vitro* ischemia model of neuronal oxygen glucose deprivation (Croslan et al., 2008). Akt was activated after NRG-1 treatment and pharmacological inhibition of the PI3K/Akt pathway prevented the NRG-1 mediated neuroprotective effect.

### 3.1 Neuroprotective effects of NRG-1 in ischemic stroke

Work from our laboratory and others demonstrated that NRG-1 reduced ischemia-induced neuronal death and inflammation in rodent focal stroke models by up to 90% (Shyu et al., 2004; Xu et al., 2004; Guo et al., 2006; Xu et al., 2006; Li et al., 2007, 2008, 2009; Guan et al., 2015; Wang et al., 2015; Simmons et al., 2016; Ji et al., 2017; Surles-Zeigler et al., 2018; Wang et al., 2018; Noll et al., 2019; Cui et al., 2023). These finding have been demonstrated by seven independent laboratories. Acute neurological and functional recovery with NRG-1 treatment was seen in multiple MCAO studies. Several of the studies continuously monitored a combination of the following physiological parameters and published the outcomes and exclusion criteria: cerebral blood flow, heart rate, respiratory rate, SpO_2_, temperature, blood pressure, blood glucose, blood gas (Shyu et al., 2004; Xu et al., 2004, 2006; Li et al., 2007; Wang et al., 2015; Ji et al., 2017; Surles-Zeigler et al., 2018; Noll et al., 2019). We showed that NRG-1 has a therapeutic window of at least 12 h after 1.5 h of ischemia and reperfusion in a transient MCAO model (Xu et al., 2006). NRG-1 administration resulted in a significant improvement of neurological function when administered 3 days following ischemia, suggesting a role in neuronal repair (Barone, 2010; Iaci et al., 2010, 2016).

Using MRI, we demonstrated that NRG-1 attenuated the expansion of the ischemic infarct into the cortical penumbra over a 48-h time span as measured by diffusion weighted imaging (DWI) and T_2_-weighted imaging (T_2_WI) following permanent MCAO (Wang et al., 2015). The DWI-defined infarcts were large at 3 h after MCAO and grew with time in the vehicle-treated control animal. Infarct expansion was prevented by a single i.a. administration of NRG-1 despite continued MCA occlusion for 48 h in the MCAO model. Subsequent studies from our lab using diffusion tensor imaging (DTI) showed that NRG-1 also protected white matter from ischemic injury (Wang et al., 2018). Preliminary findings using PWI showed that NRG-1 prevented the expansion of infarct damage into the ischemic penumbra. We developed a novel non-human primate MCAO stroke model in Rhesus macaques that will be used to examine the efficacy of NRG-1 in a gyrencephalic species using MRI, histology, neurobehavioral and other studies (Rodriguez-Mercado et al., 2012; Li et al., 2018).

Neuroprotection by NRG-1 was also observed in mice when administered i.v. (100 μg/kg) in both male and female mice. NRG-1 was neuroprotective using heterozygous NRG-1 knockout mice (NRG-1 +/−) compared with wild-type (WT) littermates (Noll et al., 2019). NRG-1−/− mice are embryonic lethal, but NRG-1 +/− mice, which have reduced NRG-1 expression (Erickson et al., 1997; Sandrock et al., 1997; Gerlai et al., 2000; Noll et al., 2019), displayed a six-fold increase in cortical infarct size compared to WT mice (Noll et al., 2019). Similar results were shown with erbB4 knockout mice following stroke where neuroprotection by NRG-1 against cerebral ischemia was prevented in the mice with erbB4 deleted in parvalbumin (PV)-positive interneurons (Guan et al., 2015).

### 3.2 BBB permeability of NRG-1

We previously examined the pharmacokinetics and ability of NRG-1 to cross the BBB after i.a. administration to rats (Li et al., 2012). Plasma maximum concentration (C_max_) values after administration was 2050 ng/mL, and time to maximum concentration (T_max_) was 0.17 h (10 min). The plasma elimination half-life (t_1/2_) was calculated at 0.14 h (8 min) and NRG-1 was undetectable after 20 min. We detected no NRG-1 in brains of control animals while animals treated with exogenous NRG-1 had detectable levels at 20 min after administration that remained constant for up to 4 h post-injection. In the brain, C_max_ was 25.0–30.2 pg./mg; T_max_ did not vary significantly from 20 min to 4 h post-NRG-1 injection. Similarly, many previous studies demonstrated that NRG-1 crosses the BBB and blood-spinal cord barrier in mice (Pan and Kastin, 1999; Kastin et al., 2004; Carlsson et al., 2011; Kato et al., 2011; Mahar et al., 2011; Rösler et al., 2011; Cui et al., 2013; Depboylu et al., 2015). Most of the injected NRG-1 reached the brain parenchyma rather than being retained in the cerebral vasculature. Systemically administered NRG-1 also induced erbb4 phosphorylation in those areas after injection, indicating that it is biologically active and reaches neuronal targets in the brain (Rösler et al., 2011).

### 3.3 Anti-inflammatory effects of NRG-1

NRG-1 demonstrated strong anti-inflammatory effects after ischemia. NRG-1 prevented macrophage/microglial activation, reactive astrogliosis, apoptosis, and cytokine expression following stroke (Xu et al., 2004, 2005, 2006; Li et al., 2007). NRG-1 treatment significantly reduced the expression of many pro-inflammatory and oxidative stress genes, including IL-1β, COX2, CD36, HSP-70, and MCP-1 (Xu et al., 2005; Simmons et al., 2016). A number of *in vitro* studies of macrophage/microglia cells including N9 microglial cells, BV-2 murine microglia, EOC murine microglia, RAW 263.7 murine macrophages, rat primary astro-microglia mixed, U937 monocytic cells showed that NRG-1 prevented the stimulation of downstream molecules by pro-inflammatory stimuli (Xu et al., 2005; Mencel et al., 2013; Simmons et al., 2016; Alizadeh et al., 2017). We showed that NRG-1 attenuated lipopolysaccharide (LPS) induced pro-inflammatory factors in microglia cells by modulating the nuclear factor kappaB (NFkB) pathway (Simmons et al., 2016). NRG-1 has been shown to associate with the NFkB-inducing kinase (NIK) that activates the alternative/non-canonical NFkB pathway to produce anti-inflammatory factors (Chen et al., 2003), Figure 1 summarizes mechanisms used by NRG-1 to protect neurons and prevent pro-inflammatory responses in ischemia and other conditions.

**Figure 1:**
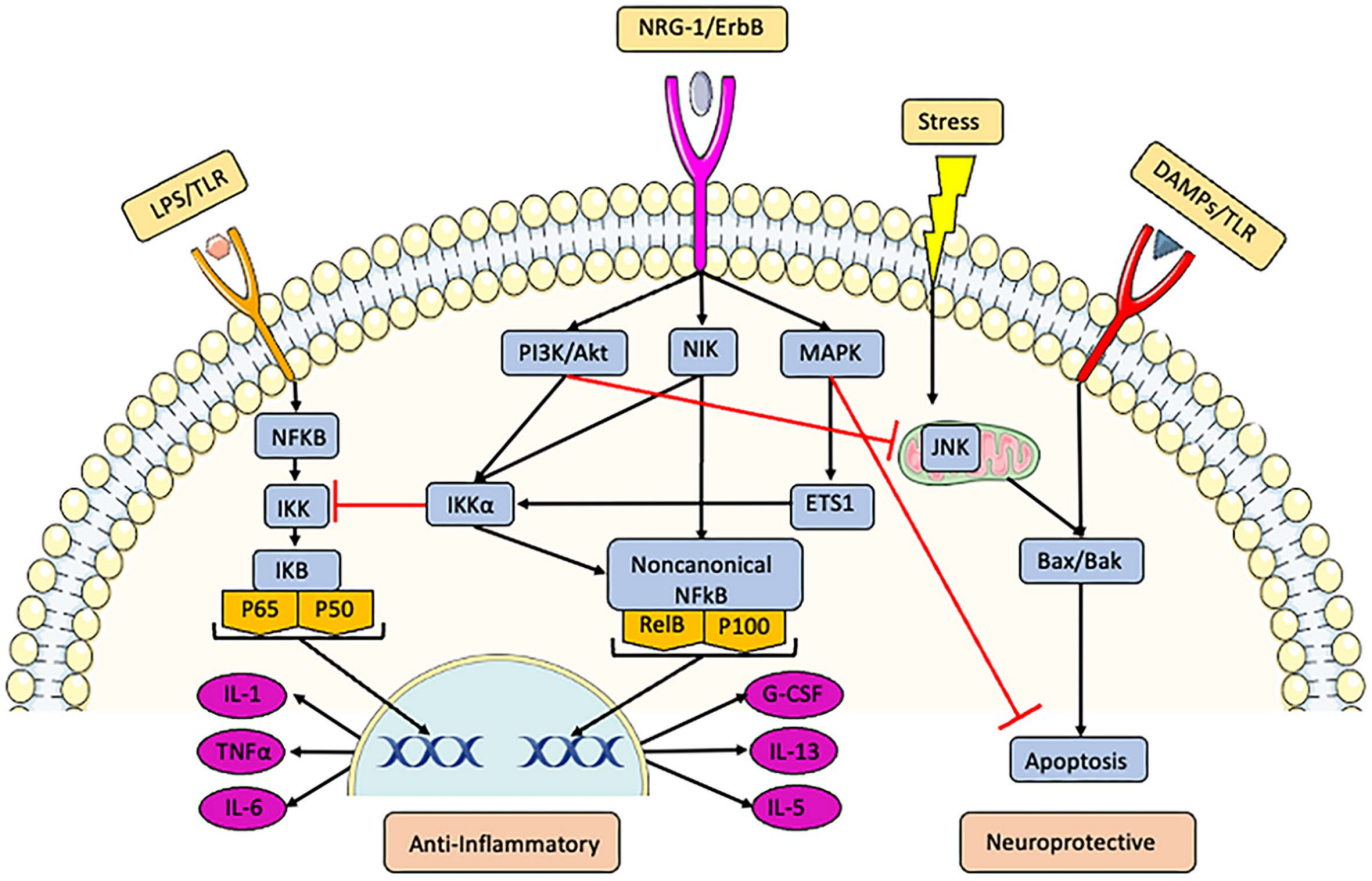
NRG-1 activates multiple pathways through erbB receptors to inhibit inflammation and initiate neuroprotection. LPS activates the common inflammation pathway through the toll-like receptor (TLR) and canonical NFkB pathways, leading to the production of pro-inflammatory factors such as TNFα, IL-6, and IL-1. Danger-associated molecular patterns (DAMPs) activate apoptosis through Bax/Bak pathways. NRG-1 acts through erbB receptors to: (1) initiate the PI3K/Akt pathway which inhibits NFkB mediated pro-inflammatory responses, (2) activate nuclear factor kappaB-inducing kinase (NIK) which also inhibits inflammation by initiating the alternative/non-canonical NFkB pathway to produce anti-inflammatory factors such as IL-13, IL-5, and G-CSF, and (3) activate the MAPK and Akt pathways to inhibit apoptosis.

### 3.4 NRG-1 improves BBB integrity

The BBB plays a critical role in stroke acutely and chronically via maintenance and resolve. After ischemic injury, there is an increase in BBB permeability leading to and an increase in edema, active resident microglia, and peripheral immune cell infiltration (Jickling et al., 2014). An efficient neuroprotectant drug could also act on the BBB, to reduce permeability and increase integrity. In a TBI mouse model, acute BBB permeability was measured NRG-1 intravenous treatment given 10 min after trauma (Lok et al., 2012). Evans blue extravasation measured 2 h after trauma exhibited 35% reduction. Studies from our laboratory showed that NRG-1 improved BBB integrity in a model of experimental cerebral malaria (Liu et al., 2018).

### 3.5 Neuroprotective effects of NRG-1 in hemorrhagic stroke

The major concern in treatment of stroke with t-PA is the increased risk of intracranial hemorrhage. Therefore, a putative neuroprotective drug would at minimum not increase cerebral bleeding, but ideally would also reduce injury following intracranial hemorrhage. Recent studies showed that NRG-1 prevented neuronal injury, improved BBB integrity and improved neurological deficits in animal models of subarachnoid hemorrhage (SAH) (Yan et al., 2017; Qian et al., 2018). ErbB4 knockdown with siRNA prevented the neuroprotective effects of NRG-1 after SAH. The tight junction proteins Occludin and Claudin-5 were significantly reduced after SAH, but NRG-1 treatment reversed both protein expressions almost back to baseline (Qian et al., 2018). Therefore, NRG-1 could potentially offer a treatment to both ischemic and hemorrhagic stroke as well as extend the therapeutic window of tPA by reducing BBB permeability and hemorrhagic transformation.

## 4 Ongoing NRG-1 clinical trials for heart failure

Recent clinical studies demonstrated the efficacy and safety of NRG-1/Neucardin in human patients with congestive heart failure (Gao et al., 2010; Jabbour et al., 2011). Recombinant human NRG-1/Neucardin was used in phase II clinical trials investigating its safety and efficacy in patients with chronic heart failure in the U.S. (ClinicalTrails.gov identifier: NCT01251406), China (Chinese Clinical Trial: ChiCTR-TRC-00000414), and Australia (Australian New Zealand Clinical Trials Registry: ACTRN12607000330448). Patients received NRG-1/Neucardin at a dose of 0.3–1.2 μg/kg/day or placebo i.v. for 10 days, in addition to standard of care therapies. Acutely, cardiac output increased by 30% during a 6-h NRG-1/Neucardin infusion (Jabbour et al., 2011). During a follow-up period 11–90 days after study initiation, NRG-1/Neucardin significantly improved cardiac function in patients and the effective doses were shown to be safe and tolerable (Gao et al., 2010). Two additional clinical trials to determine the ability of NRG-1/Neucardin to improve cardiac function after heart failure have been initiated in the U.S. (ClinicalTrails.gov identifiers NCT02664831; NCT01258387).

NRG-1/Cimaglermin is the full-length extracellular domain of NRG-1β3, also known as glial growth factor 2 (GGF2), which has been used in clinical development for chronic heart failure. A phase 1, double blind, placebo-controlled, single ascending dose study examined the safety, tolerability, and exploratory efficacy of intravenous infusion of recombinant NRG-1/Cimaglermin alfa, in patients with heart failure (Lenihan et al., 2016). Forty patients with symptomatic heart failure were randomized to NRG-1/Cimaglermin or placebo in 7 ascending dose cohorts (0.007 mg/kg, 0.021 mg/kg, 0.063 mg/kg, 0.189 mg/kg, 0.378 mg/kg, 0.756 mg/kg, and 1.512 mg/kg). There was a dose-dependent improvement in left ventricular ejection fraction lasting 90 days following infusion. NRG-1/Cimaglermin treatment was generally tolerated except for transient nausea and headache and a dose-limiting toxicity was noted at the highest planned dose. There were no acute adverse effects leading to termination of drug infusion. A number of additional studies have shown that NRG1 can repair the heart after myocardial infarction, cardiomyopathy, atherosclerosis, and other cardiovascular diseases (Hertig et al., 1999; Geisberg and Lenihan, 2011; Galindo et al., 2014; Wang et al., 2022).

## 5 Conclusion and discussion

The failed translation from animal experimental stroke studies to clinical studies has created a great deal of pessimism over the neuroprotection hypothesis. Although animal stroke research has not directly yielded new clinical drugs, it has provided important mechanistic insights into the complex pathophysiology of ischemic stroke which will pave the way for future therapies. NRG-1 has demonstrated promise as an acute neuroprotectant and anti-inflammatory agent in preclinical animal stroke models. These effects have been seen in multiple cerebral injury models, using various administration methods with clinically relevant doses, and verified by several independent laboratories. Importantly, NRG-1 is a peptide that can cross the intact BBB in animal models and activate its target receptors in the brain. Other neurotrophic and neuroprotective factors, such as glial-cell line derived growth factor (Kastin et al., 2003b), platelet derived growth factor (PDGF) (Kastin et al., 2003a), TGFα (Pan et al., 1999) and transforming growth factor β (TGFβ) (Kastin et al., 2003c) do not cross the BBB, therefore NRG-1 is an intriguing candidate for therapeutic treatment of CNS disorders due to its accessibility to the brain parenchyma. Taken together data from our laboratory and others show that NRG-1 has fully or partially fulfilled many of the STAIR criteria including, 1) randomized and blinded studies; 2) permanent occlusion and transient ischemia models; 3) histological outcomes assessment (along with MRI); 4) defined time window; 5) efficacy in two or more laboratories; 6) consideration of clinically relevant administration route; and 6) consideration of sex difference. Additionally, NRG-1 clinical trials have been completed demonstrating safety and efficacy in human patients with heart failure using similar doses to those in preclinical stroke studies, potentially increasing the odds of success in ischemic stroke trial. Future animal and human studies will consider other factors including age differences, co-morbidity and pharmacological profile for NRG-1 treatment.

The STAIR criteria recommend that clinical trials with a neuroprotectant be designed as adjuvant treatment with tPA (Albers et al., 2011; Jovin et al., 2016). Clinical studies specifically testing effects of neuroprotective agents in addition to tPA and/or EVT are limited (Vos et al., 2022). We propose to use a combination therapy approach with NRG-1 alongside thrombolysis with tPA or EVT, which could produce synergistic protective effects through different mechanisms. Combination therapy with NRG-1 could potentially extend the current therapeutic time window of tPA and reduce adverse effects such as intracerebral hemorrhage. Preclinical studies are underway in our laboratory to determine the safety and efficacy of NRG-1 when used in conjunction with tPA. Based on studies of NRG-1 using models of subarachnoid hemorrhage, NRG-1 could potentially offer a treatment to both ischemic and hemorrhagic stroke as well as potentially extend the therapeutic window of tPA by reducing BBB damage and hemorrhage. The role of NRG-1 in reducing BBB damage and increasing integrity may also play a part in its anti-inflammatory role. *These findings suggest that NRG-1 treatment would not induce hemorrhagic transformation after ischemia and could protect patients from t-PA mediated toxicity*.

ENACT and ESCAPE-NA1 provided some evidence that neuroprotection in stroke patients is indeed feasible. Interestingly, tPA was shown to reduce plasma concentration of nerinetide, and it was suggested that nerinetide contains amino acid sequences known to be cleaved by plasmin resulting in a possible drug–drug interaction (Hill et al., 2020). This further confirms the need to conduct neuroprotection along with thrombolysis and/or EVT. Subsequent analysis of the ESCAPE-NA-1 trial indicated that infarcts in a new territory (INT), are common and known complications of EVT for acute ischemic stroke associated with poorer outcomes (Singh et al., 2023). INT is defined by an imaging-proven infarct in a vascular territory outside that of the original target occlusion before EVT. These are caused by distal microemboli that travel downstream from the EVT site. Thus, strategies to reduce INTs are a treatment target for improving outcomes for patients with stroke due to EVT. In the ESCAPE NA-1 trial, 10% of the subjects were found to have INT on postprocedural MRI (Singh et al., 2023). The follow-up ESCAPE-NEXT clinical trial (NCT04462536) will examine the efficacy of nerinetide on the incidence of INT in subjects who undergo endovascular treatment. We propose to use INT frequency as an outcome measure of NRG-1 efficacy following ischemic stroke in combination with EVT and/or tPA to protect the brain from distal micro emboli that could lead to INT.

**Figure 2:**
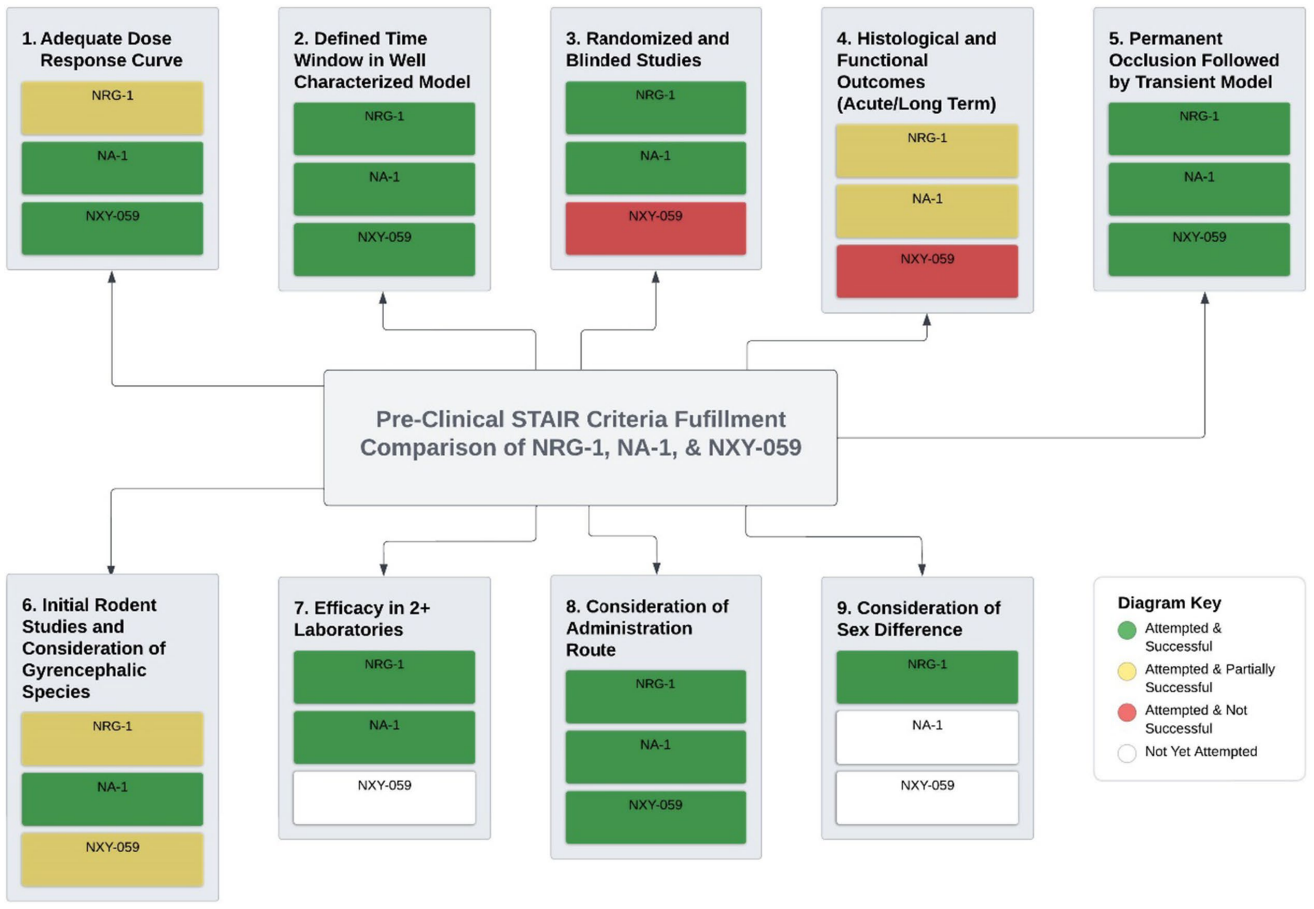
NRG-1 potential as a clinical treatment for stroke. Findings from our laboratory and others further indicate that NRG-1 has fulfilled many STAIR criteria that already place it in advantage over NXY-059 and comparable to nerinetide that have reached clinical trials NRG-1 has shown success in completing of the STAIR criteria where NXY-059 did not, robust neuroprotection in rodent models, blinding and randomization, extended time windows in clinically relevant stroke models and reproducibility in multiple laboratories. Nerinetide has shown some hope for neuroprotective strategies for stroke and NRG-1 is on track to similarly fulfill the STAIR criteria.

Taken together, we demonstrate that NRG-1 has outstanding potential as a clinical treatment for stroke. Findings from our laboratory and others further indicate that NRG-1 has fulfilled many STAIR criteria that already place it in advantage over NXY-059 and comparable to nerinetide that has reached clinical trials (Figure 2). NRG-1 has shown success in completion of the STAIR criteria where NXY-059 did not, robust neuroprotection in rodent models, blinding and randomization, extended time windows in clinically relevant stroke models and reproducibility in multiple laboratories (Savitz, 2007). Nerinetide has shown some hope for neuroprotective strategies for stroke and NRG-1 is on track to similarly fulfill the STAIR criteria. In consideration of these findings, we suggest that NRG-1 move toward clinical trials based on lessons learned from previous stroke clinical trials, including NXY-059 and nerinetide studies.

## Author contributions

JN: Conceptualization, Formal analysis, Investigation, Writing – original draft, Writing – review & editing. AS: Formal analysis, Investigation, Writing – review & editing. GF: Investigation, Writing – review & editing, Data curation. BF: Investigation, Writing – review & editing, Conceptualization, Formal analysis, Funding acquisition, Resources, Supervision, Writing – original draft.
